# Path Planning and Obstacle Avoidance of Formation Flight

**DOI:** 10.3390/s25082447

**Published:** 2025-04-12

**Authors:** Yi-Sin Yang, Jih-Gau Juang

**Affiliations:** Department of Communications, Navigation and Control Engineering, National Taiwan Ocean University, Keelung 20224, Taiwan; yang.ysin610@gmail.com

**Keywords:** UAV, path planning, obstacle avoidance, formation flight, A* algorithm, reinforcement Q-learning, genetic algorithm, leader–follower algorithm

## Abstract

This study applies path planning and obstacle avoidance to drone control for conducting riverbank inspections. Given that the river’s surrounding environments are often windy and filled with overgrown branches and unknown obstacles, this study improves path planning and obstacle avoidance to enable drones to complete inspection tasks using the planned optimal route. Multiple drones are used for larger-scale areas to reduce time consumption and increase efficiency. Regarding path planning, the A* algorithm is improved using a grid-based approach. For obstacle avoidance, depth cameras are installed on the drones, and the obtained images are processed by reinforcement Q-learning with a genetic algorithm to navigate around obstacles. Since maintaining formation is necessary during task execution, the leader–follower method of formation flight ensures that multiple drones can complete inspection tasks while maintaining formation.

## 1. Introduction

Unmanned aerial vehicles (UAVs) are commonly used in various research fields, such as the unmanned multi-rotor drones in this study. Equipping drones with specialized tools can replace traditional labor, saving time and cost while enhancing safety. In recent years, drones have increasingly replaced humans in various tasks, including water quality sensing in different environments, aerial photography, agricultural and forestry management, logistics and transportation, environmental protection, disaster relief, national defense, and public infrastructure inspections. In these scenarios, effective path planning and obstacle avoidance can minimize unnecessary inefficiencies, and multiple drones may be required for more effective task execution in larger areas. With the advancement of technology, drones are being utilized in an increasingly wide range of applications, making them a popular topic of discussion. Drones can be categorized into unmanned airplanes, helicopters, and multi-rotor aircraft. Unmanned airplanes have fixed wings, allowing longer flight times and good wind resistance. However, they require sufficient runway length for takeoff and landing and cannot quickly maneuver around obstacles. Unmanned helicopters and unmanned multi-rotor aircraft fall under the category of rotorcraft. Unmanned helicopters can take off and land vertically, offering high flexibility and the ability to hover for extended periods. Despite these advantages, their hardware structure is more complex, making them more challenging and expensive to operate. Unmanned multi-rotor aircraft, like unmanned helicopters, can take off, land vertically, and hover in place. Although multi-rotor aircraft have shorter battery life and slower speeds than the other two types, they can be equipped with various tools to enhance their efficiency and are easier to operate. Therefore, unmanned multi-rotor aircraft are the most suitable choice in this study. The main objective of this study is to use drone technology to replace traditional human labor. Some river areas are remote, dangerous, or densely populated, making comprehensive garbage inspections difficult. Over time, this can negatively impact the ecosystem. To reduce human labor and ensure safer and faster task execution, this study first plans safe and optimal paths with multiple waypoints. This allows drones to perform inspections accurately. Then, the obstacle avoidance system enables real-time evasion of obstacles, ensuring flight safety. Additionally, using multiple drones in broad areas allows for more extensive inspections within a fixed time frame, achieving maximum efficiency.

One of the primary considerations before executing any drone task is path planning. Path planning has been developed for a long time, and numerous methods are available [1]. Among them, sampling-based algorithms like Rapidly-exploring Random Tree (RRT) are the most common. However, the paths and computation times are often not optimal due to their random sampling nature. To address this, H. Yan and X. Fu increased the weight of target points in the RRT search area to reduce the time required for drone path planning. They combined RRT with a Kalman filter to predict obstacle movement trajectories and used B-Spline curves to optimize the route [2]. In three-dimensional space, H. Yin and L. Zhang combined the features of RRT and A*, first allowing RRT to sample the environment and then using these sampled points for A* path planning, resulting in shorter times than the original A* and lower path costs than RRT [3]. Since RRT cannot obtain safe paths in dynamic environments, G. Wang, C. Jiang, G. Tao, and C. Ye proposed improvements to RRT through target bias sampling and parent node reselection. Target bias sampling extends sampled points toward the target, while parent node reselection removes redundant nodes to shorten the path. They adjusted function weights using fuzzy rules in the Dynamic Window Approach (DWA) to reduce iterations and computation time while maintaining safe distances from obstacles and improving environmental adaptability. Their experiments verified the feasibility of this hybrid algorithm for path planning and real-time obstacle avoidance [4]. Over the years, RRT has been developed into many branches, including bidirectional RRT (Bi-RRT), which extends nodes from both the start and end points. Although Bi-RRT grows bidirectionally, it retains the random growth characteristics of RRT. Z. Zhang et al. proposed a Bi-RRT*-Smart method, optimizing paths to be shorter and safer [5]. Among search-based algorithms, D*Lite [6] and A* [7,8] are commonly used and often improved by combining them with other algorithms. With the development of algorithms, bio-inspired heuristic methods based on biological behaviors have emerged, such as the Grey Wolf Optimizer (GWO) algorithm [9]. Due to their ability to yield optimal solutions, they are also used in path planning. H. Liu applied Particle Swarm Optimization (PSO) in the crossover and mutation steps of genetic algorithms (GA) to obtain better parameters and improved results [10]. In recent years, new algorithms have been developed, such as the Bat Algorithm (BA), characterized by environmental solid adaptability but prone to local optima. To avoid premature convergence to local optima and increase the accuracy of global optimization, Y. Jiang, Y. Wu, and M. Wang added weight parameters to the updated learning rule [11]. In complex three-dimensional spaces, X. Zhou, F. Gao, X. Fang, and Z. Lan integrated the Artificial Bee Colony (ABC) algorithm with BA, resulting in faster and safer paths than BA alone [12]. In dynamic environments, Ajeil FH, Ibraheem IF, Humaidi AJ, Khan ZH, et al. proposed changing sound frequencies to avoid obstacles [13] successfully. Yuan, X., Yuan, X., and Wang, X. enhanced the BA with a logarithmic decreasing strategy and Cauchy perturbation to improve searchability, combining it with DWA for more efficient operation in complex environments [14].

After completing the initial path planning, it is essential to consider unforeseen situations, making obstacle avoidance an indispensable consideration [15]. Besides the commonly used Artificial Potential Fields (APF) [16,17,18], reinforcement learning methods have also started to be employed for obstacle avoidance [19]. Since reinforcement learning requires data input, cameras are often used in experiments, with the images captured by the camera serving as the basis for obstacle avoidance. N. S. Manikandan, G. Kaliyaperumal, and Y. Wang compared three deep reinforcement learning methods—DQN, DDQN, and D3QN—using camera input [20]. J. Ding et al. [21], I. Asante, L. B. Theng, M. T. K. Tsun, H. S. Jo, and C. McCarthy [22], and A. Singla, S. Padakandla, and S. Bhatnagar [23], also used cameras with different reinforcement learning approaches, achieving good results. Because cameras are relatively cheaper than LiDAR, many researchers use cameras as tools for obstacle avoidance, route calculation, and environmental inspection [24,25]. The integration of decision-making and motion planning frameworks for enhanced oscillation-free capability was proposed in [26]. The control process can make proper, safe, and continuous decisions and planning for autonomous vehicles. In [27], the authors presented a high-precision tracking control problem of UAVs under external disturbances using a dual-layer robust sliding mode control strategy. A data-driven approach was employed to construct a PSO-LSTM (Long Short-Term Memory) model for vertical take-off and landing position error data. The PSO-LSTM can improve its predictive performance on new data. When the number of drones increases, it becomes necessary to implement control measures to prevent potential loss of control. As drone technology advances, formation flight has gained increasing attention [28]. The leader–follower approach in formation control is widely applied, with R. Guan and G. Hu [29] and L. Ma, D. Meng, X. Huang, and S. Zhao [30] incorporating cameras to maintain formation. A formation model combining an improved particle swarm optimization algorithm and a generative route optimization method based on an improved Douglas–Peucker algorithm was proposed in [31]. The improved model performs better in terms of accuracy and stability of formation target allocation and route planning. In a multiple-vehicle formation structure, intervehicle collision should be paid attention to. In [32], the authors presented a switch strategy for multiple vehicles while considering intervehicle collision avoidance. By employing the artificial potential field, the guidance principle can program the real-time attitude reference for vehicles by using the security intervehicle distance while achieving the path-following task. Research on formation control primarily focuses on two issues: formation tracking and formation shape generation. Formation shape generation involves guiding multiple unmanned vehicles to designated target positions through specific design methods, forming a predetermined formation [33]. Our study is divided into three parts. First, the path planning is improved upon by the grid-based A* algorithm to perform offline path planning calculations, yielding better results than the traditional A* method. The second part addresses obstacle avoidance, incorporating dynamic obstacles and utilizing reinforcement learning for better adaptability. The Q-learning algorithm with GA is chosen for experiments in complex environments due to its superior learning capabilities. Finally, the results from the first two parts are integrated; a formation flight method is used to control the drones’ positions after planning the flying route. The leader–follower method is applied to formation flight, which explains the relationships between UAVs and how to control multiple UAVs. Path planning, obstacle avoidance, and formation flight are applied to actual UAVs and conducted in real environments. The computations are processed on the onboard computer and combined with external sensors to enable UAVs to perform river inspections effectively. A table listing comparisons of different methods is shown in Table 1.

## 2. UAV System

**Figure 1:**
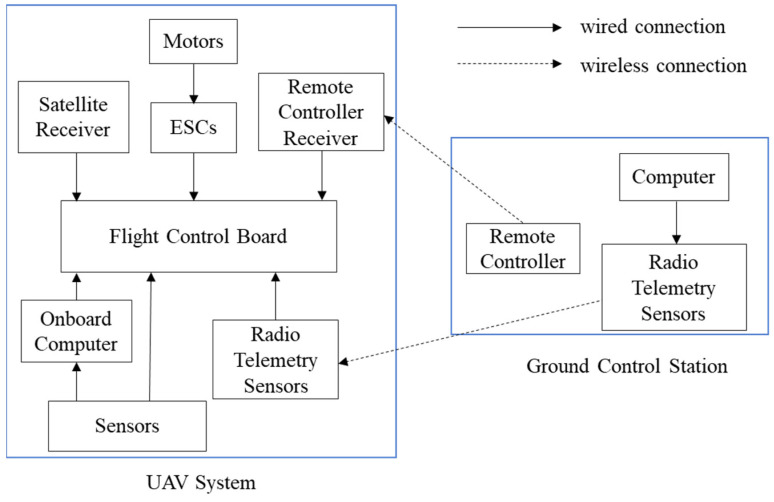
UAV flight system architecture.

This study categorizes the unmanned aerial system into the ground control station and the drone system. The ground system mainly includes a computer, a drone remote controller, and radio telemetry sensors. At the ground control station, signals can be sent to the drone through the computer, remote controller, and radio telemetry sensors for the drone’s response status. The most crucial part of the drone system is the flight control board, which connects to various sensors on the drone, including the satellite receiver, motors, battery, onboard computer, remote control receiver, and electronic speed controllers (ESC). Figure 1 provides a schematic diagram of the drone flight system architecture.

The UAV is used as the primary operational platform. The flight control board plays a crucial role in drone flight. As an independent open-source project, it effectively integrates the functions of connected sensors into flight operations. The flight control board has built-in accelerometers, barometers, gyroscopes, magnetometers, and dual altimeters. These sensors enhance the drone’s stability during flight and provide precise positioning. Additionally, the remaining ports on the flight control board allow for the attachment of additional components, offering further development opportunities for the control board. The two flight control boards used in this study were Pixhawk-4 and Pixhawk-6X (Holybro, Hong Kong, China) [34].

**Figure 2:**
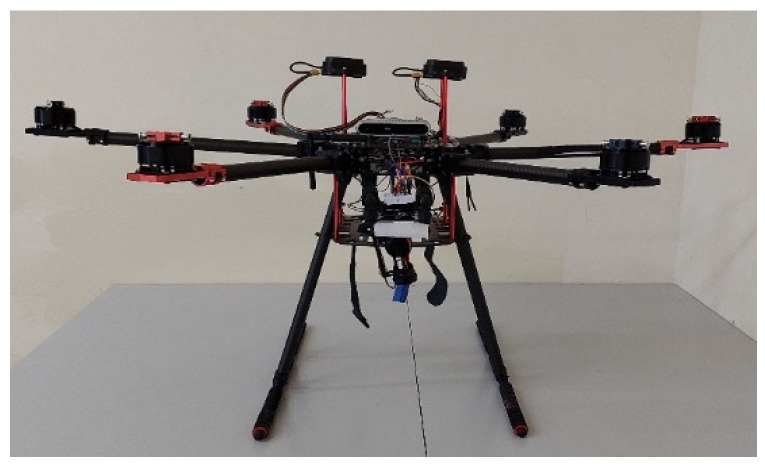
Six-rotor UAV.

**Figure 3:**
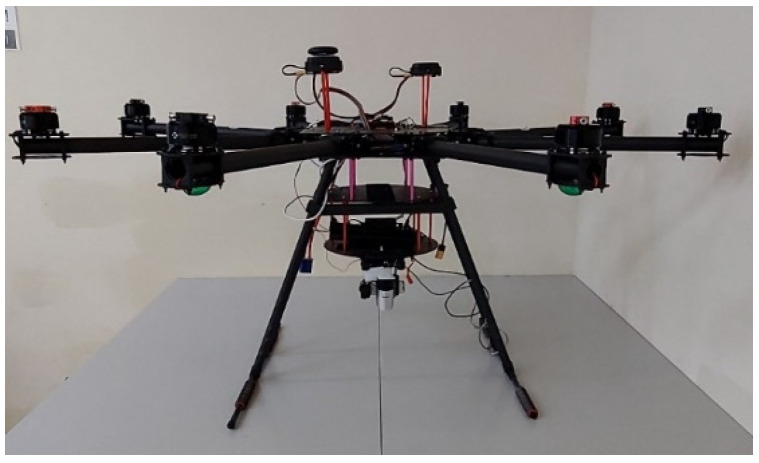
Eight-rotor UAV.

In this study, we set up multi-rotor drones for experimentation. Compared to quadcopters, six-rotor and eight-rotor drones offer better stability in strong winds and are less likely to crash due to the loss of a single rotor in emergencies. Therefore, we selected two six-rotor drones and one eight-rotor drone as the vehicles for our tasks. Figure 2 shows the first custom-built six-rotor. Figure 3 presents the eight-rotor UAV.

**Figure 4:**
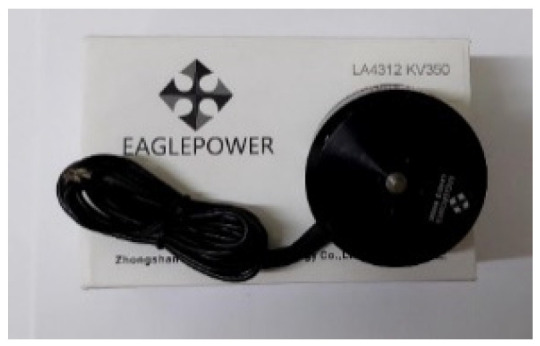
LA4312 350KV brushless DC motor.

**Figure 5:**
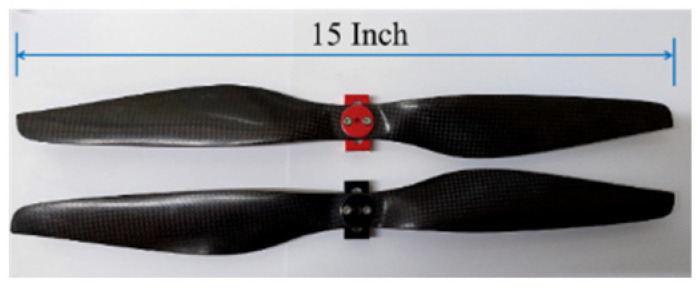
Carbon fiber propeller, 15-inch.

**Figure 6:**
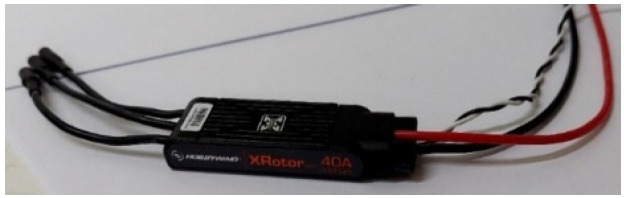
Hobbywing XRotor PRO 40A 3D.

The output of the drone’s lift power system consists of several motors, electronic speed controllers (ESCs), and propellers. A general principle for selecting motors is known as “KV”. The KV value represents the motor’s RPM (revolutions per minute) per input voltage. A lower KV value indicates a lower RPM for the same voltage, resulting in higher efficiency and allowing the use of enormous torque and propellers, which benefits the drone. Therefore, this study used LA4312 350 KV motors (Eaglepower, Zhongshan, China) (Figure 4). The propellers must be matched with the motors. Higher KV motors require smaller propellers, while lower KV motors can use larger propellers. Based on the drone’s size and weight, 15-inch carbon fiber propellers were selected, as shown in Figure 5. Electronic speed controllers (ESCs) play a crucial role between the motors and the flight control board, receiving commands from the control board to drive the motors. This study used Hobbywing XRotor PRO 40A 3D ESCs (Hobbywing Technology, Shenzhen, China), as shown in Figure 6.

**Figure 7:**
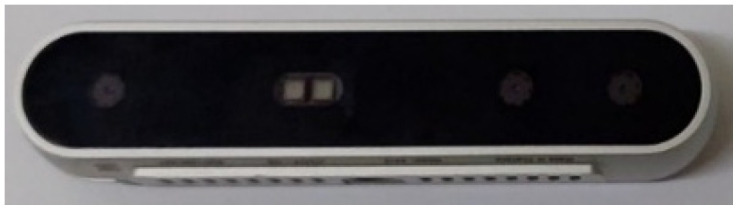
Intel RealSense D415.

Once the motor and propeller combination are determined, the choice of battery will affect the drone’s flight time. Since the battery is the drone’s power source, selecting an appropriate battery is crucial. Although a battery with a higher capacity can increase flight time, its weight also increases, which adds to the drone’s load. In this study, a 16,000 mAh rechargeable lithium battery, weighing about 2 kg, was used for the six-rotor drone, allowing the drone to fly for approximately 20–25 min. A 22,000 mAh rechargeable lithium battery, weighing around 2.5 kg, was used for the eight-rotor drone. Despite the increased capacity, the flight time remains around 20 min due to the heavier drone. We equipped the drone with an additional depth camera to meet this study’s real-time obstacle avoidance needs. The images captured by the camera are used to avoid obstacles. Depth cameras are effective not only indoors but also outdoors. This study used the Intel RealSense Depth Camera D415 [35] as the primary obstacle avoidance tool, as shown in Figure 7. The D415 Camera is powered via USB and consists of a depth sensor, an RGB sensor, and an infrared projector, offering a tightly focused field of view.

**Figure 8:**
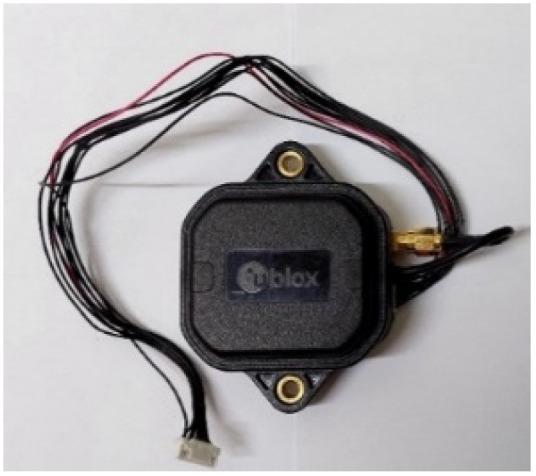
PMGN.

**Figure 9:**
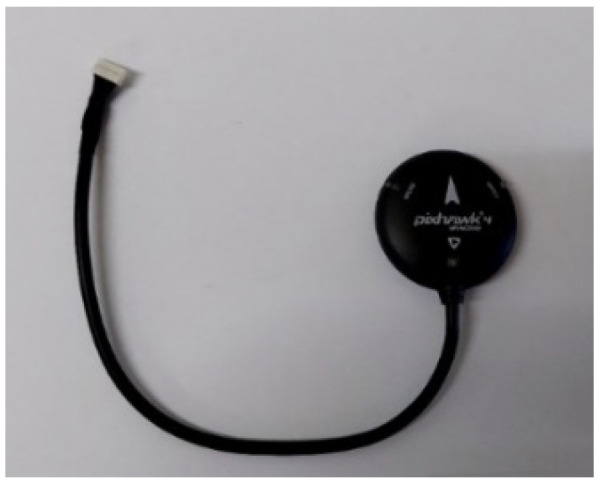
Neo-M8N.

To enhance the accuracy of drone positioning, in addition to the GPS component U-blox Neo-M8N on the Pixhawk 4 flight control board, we added two satellite receivers. The PMGN1 is a module with RTK positioning accuracy, capable of receiving signals from four GNSS systems (GPS, GLONASS, Galileo, and BeiDou). Using two PMGN1 modules together ensures that the positioning error between the drone and the preset location is less than 30 cm, achieving high accuracy. The PMGN1 satellite receiver is shown in Figure 8. Another flight control board used in this study is the Pixhawk 6x. During experimentation, it was found that the Pixhawk 6x often had connectivity issues with the high-precision PMGN1. Therefore, the Neo-M8N was used as the positioning module for two drones. Although its accuracy is lower than the PMGN1, it is widely used and highly compatible with drone positioning systems. Its module is shown in Figure 9.

Due to the requirements of our study for path planning, obstacle avoidance, and other crucial tasks, which involve complex and demanding computations for the drone and flight control board, we needed to use an onboard computer to handle these operations. Additionally, a depth camera requires sufficient computational power to meet its performance needs. Therefore, we used the NVIDIA Jetson AGX Xavier (NVIDIA, Santa Clara, CA, USA) as the onboard computer for the drone. This computer can smoothly handle complex programs and provide real-time updates during missions, showcasing high performance while consuming minimal energy. It is an ideal tool for this research. The NVIDIA Jetson AGX Xavier and its specifications can be found in [36].

**Figure 10:**
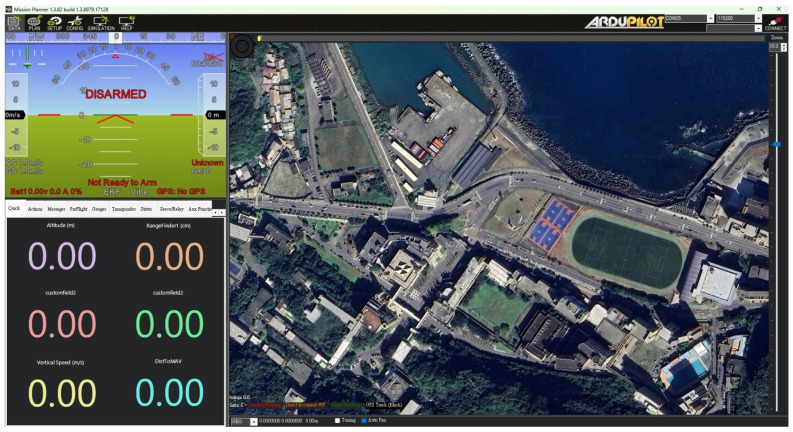
Mission planner operating platform.

**Figure 11:**
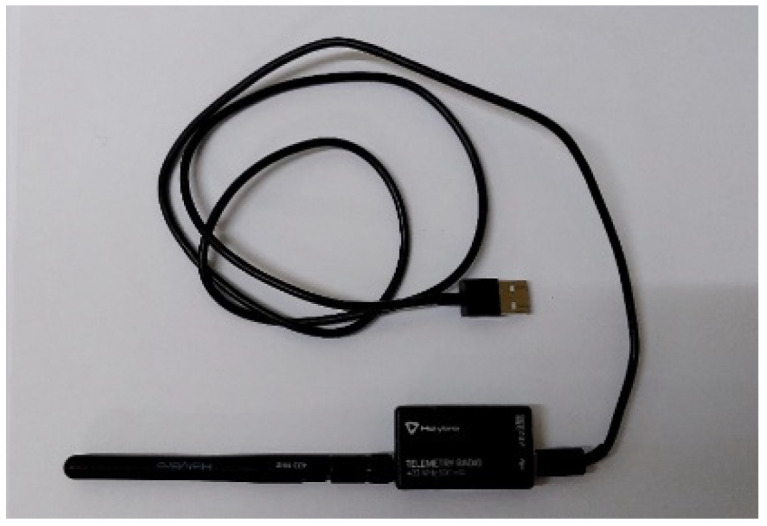
Radio telemetry.

Mission Planner [37] is our study’s primary ground station operation platform. It is an open-source application developed by Ardupilot. By connecting this platform with our flight control board, we can obtain a wealth of information on the interface, such as the drone’s real-time position, altitude, and angle. It also allows us to identify and address any issues within the flight control board and check for anomalies in GPS positioning. The platform records all data, as shown in Figure 10. The platform’s built-in functions can be integrated with the drone’s GPS, enhancing the accuracy of the positioning system and improving precision. The drone can be calibrated on-site in unfamiliar environments for more accurate flight parameters. Also, waypoints can be set on the map, allowing the drone to follow commands autonomously. Both the drone and the ground station use radio telemetry. Since we use more than one drone, and radio telemetry can only be used in a one-to-one situation, using the same frequency band would prevent us from simultaneously receiving information from all drones. To address this, we used radio telemetry frequencies of 433 MHz and 915 MHz. By adjusting the frequencies, we can ensure that even if there are other telemetry radios on the same frequency, we can still receive information from the drones without interference. Figure 11 shows the radio telemetry unit.

## 3. Path Planning

This study aims to set points according to river locations, enabling the drone to complete tasks quickly and with the shortest path. The experiment is conducted in a grid-based environment, meaning only points on the grid are traversable, and there will be no curved paths. Any moving object requires path planning, whether it is an autonomous vehicle, a robot, or a drone. Path planning involves finding a trajectory from the starting point to the endpoint within a traversable area while avoiding obstacles. Path planning can be categorized into global path planning and local path planning. It can also be classified based on the environment into static and dynamic path planning, and further into known and unknown environments.

### 3.1. Non-Grid Method

**Figure 12:**
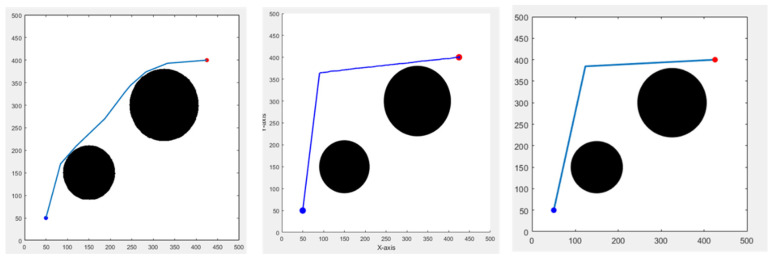
Different algorithms result in a non-grid environment, blue and red circles are the start and end points, respectively.

First, we set up obstacles in an environment of 500 m in length and width. The Bi-RRT*-Smart takes a shorter time to find a path due to its RRT characteristics. It adds two-way simultaneous search and path optimization, allowing this method to keep the time short and the path not too long at the same time. The other two bionic algorithms perform well when obstacles appear in the environment; the effect is good. The calculation time will inevitably be a bit long because they are bionic meta-heuristic algorithms. However, the time is still within 5 s. Although the path length may be somewhat longer, it can usually be used for global path planning. The results are shown in Figure 12, and the data are presented in Table 2. Figure 12, from left to right, displays the results of the Bi-RRT*-Smart, BA (Bat Algorithm), and GWO (Grey Wolf Optimizer) algorithms [9].

### 3.2. Grid Method

Among grid methods, the A* algorithm is relatively more straightforward in its mathematical formulation and easier to understand than other algorithms [38]. This simplicity and comprehensibility have led to its widespread use and frequent improvements. Therefore, A* for planning in a grid-based environment is used in this study. In addition to the traditional A* algorithm, two improved versions based on A* are applied for comparison, evaluating the effectiveness of all three methods.

#### 3.2.1. A* Algorithm

**Figure 13:**
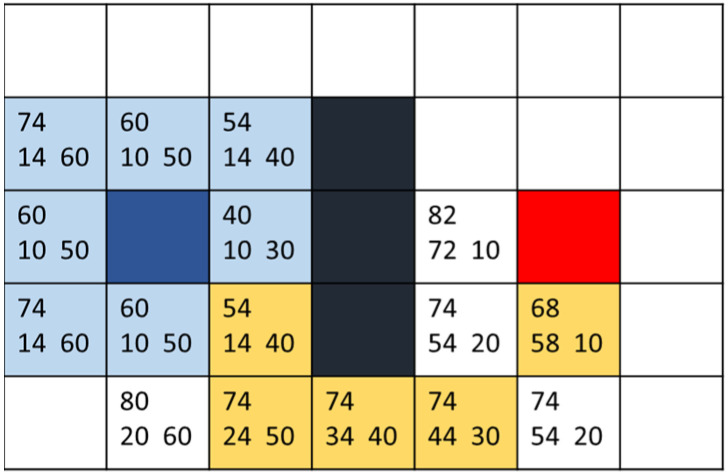
A* schematic diagram of selected path.

The A* search algorithm is often used to calculate the best path-planning method. This algorithm tries to find the lowest path cost when multiple nodes are on the graphics plane. It evolved from the Dijkstra algorithm. Dijkstra calculates all the points in the environment, thus wasting much performance. A* improved this point and proposed a set of formulas only to calculate the places where it is possible to go. The formula for the evaluation function is as follows:

(1) $$F\left(n\right)=G\left(n\right)+H\left(n\right)$$

where *n* is the current node, *G*(*n*) function is the distance from the starting point to the actual movement of the current node, *H*(*n*) is the estimated distance from the current node to the endpoint, and *F*(*n*) is the sum of the two. In performing path planning, the cost values from the starting point to the eight directions are calculated, then the mesh with the smallest value is selected to recalculate the cost values around it. The above steps are repeated until the current node contains endpoints and the path is found with the help of the parent list. Based on the values of the *F*(*n*) function, neighboring points are explored, and the *H* and *G* values are calculated for each. The lower the total cost function, the higher the priority for the search. This results in generating the optimal path with the minimum cost. As shown in Figure 13, the number in the lower left corner of each square represents the cost *G* of the point from the blue start point (usually calculated by length). In comparison, the number in the lower right corner represents the movement cost *H* to the red endpoint (considering only movements at right angles and ignoring black obstacle boxes). The number in the upper left corner is the sum of the two movement costs, *F*. The yellow path denotes the final route.

Conventional A* uses the Manhattan distance as the cost function for estimating *H*(*n*). There are three common cost functions for A*, as shown below.

1. Manhattan distance

(2) $$H\left(n\right)=\left|X_{n}-X_{end}\right|+\left|Y_{n}-Y_{end}\right|$$

2. Euclidean distance

(3) $$H\left(n\right)=\sqrt{{\left(X_{n}-X_{end}\right)}^{2}+{\left(Y_{n}-Y_{end}\right)}^{2}}$$

3. Chebyshev distance

(4) $$H\left(n\right)=max(\left|X_{n}-X_{end}\right|,\left|Y_{n}-Y_{end}\right|)$$

The traditional A* search algorithm considers eight possible directions for searching. In the search program, the nodes that need to be calculated in the environment are stored in the Open List. Then, the already calculated nodes are placed in the Close List. When encountering a node that does not meet the desired criteria, the algorithm checks its eight neighboring nodes. If these neighboring nodes are not in either of the lists, they are added to the Open List. If an adjacent node is already in the Open List but has a higher cost, it gets updated to a lower cost. If a node is in the Close List, the algorithm checks if using this node for a new path is cheaper. If so, it is swapped with a node in the Open List, ensuring that the chosen nodes always represent the lowest cost.

#### 3.2.2. Improved A* Algorithm

**Figure 14:**
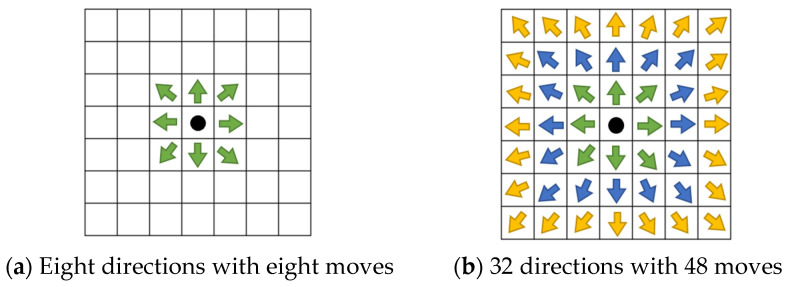
Search schematic of A* and proposed improvement A*, the green, blue, and yellow arrows represent the directions in layer-1, layer-2, and layer-3, respectively.

In this study, based on [39,40], we improved the A* algorithm and expanded the search range to three layers, increasing the number of feasible directions from the original 8 to 32. This modification allows the proposed method to reach 48 adjacent cells, as shown in Figure 14. The expanded search range significantly broadens the search area.

The *H* function is a crucial part of the A* algorithm. Different cost functions can lead to the discovery of other paths. Many scholars have experimented with adjusting the weights of the *H* function, achieving notable results. However, these experiments typically do not restrict the search range; instead, they focus on adjusting weights to make the path smoother. Consequently, there is limited research on grid-based searches.

**Figure 15:**
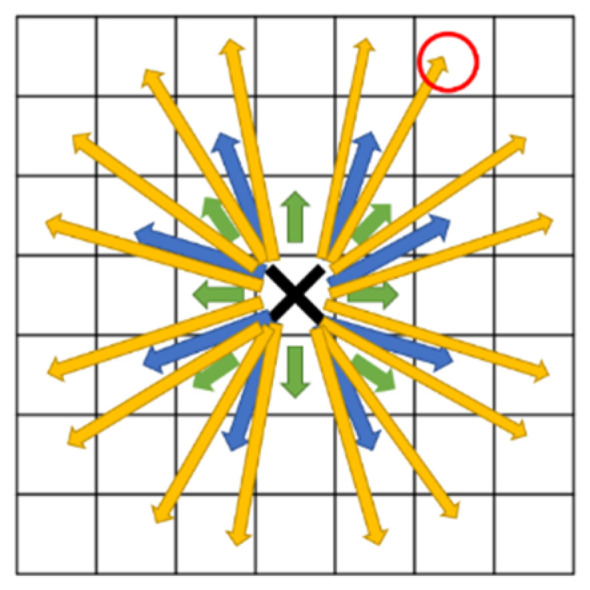
Schematic of 32 directions.

In this study, based on the traditional A* algorithm, the improved A* optimizes the following two points:

1. Uniform Movement Cost: The first improvement involves making the movement cost to the 48 adjacent grid cells identical. This change ensures that nodes further away from the original node are not excluded due to higher movement costs, leading to a faster overall path.
2. Obstacle Avoidance: The second improvement is that when selecting an optimal node, if the path encounters an obstacle, that node is not used, and the algorithm must choose another. As illustrated in Figure 15, if the black cross represents the original node (0, 0) and the red circle represents the initially chosen node (2, 3), the algorithm can directly reach this point according to the first rule. However, if any of the positions (1, 1), (1, 2), or (2, 2) contain obstacles, the red circle node cannot be selected. In real-world scenarios, traveling along a supposedly safe route and colliding with an obstacle is impossible.

The improved A* algorithm can provide faster and better path planning by adding these improvements.

### 3.3. Comparison of Simulation Results

For the three methods of A*, improved A* [39,40], and improved A* proposed in this study, their path lengths and time spent are compared in different scenarios, and the three methods mentioned above are hereafter referred to as A*-8 directions, A*-16 directions, and A*-32 directions.

#### 3.3.1. Environment with Obstacles

**Figure 16:**
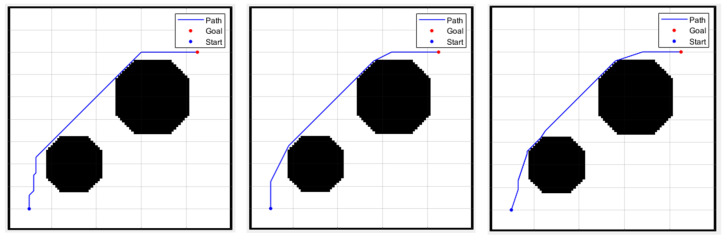
From left to right: A*-8 directions, A*-16 directions, A*-32 directions.

A specially designed scenario with the same environment size and obstacles as in the non-grid environment was created. Due to the fact that the grid environment’s node positions are integers while the non-grid environment can have decimal values, there will be slight differences in the shape of obstacles. Apart from the differences in the environment composition, all other settings are identical. The simulation results of different A* methods with different directions are shown in Figure 16. Table 3 shows that as the number of directions increases, there is a significant difference in time and distance between the traditional A* algorithm and the improved A* algorithm. Additionally, from the resulting figures, it is evident that having more directions provides more options and results in smaller turning angles.

In this scenario, we have adjusted the grid size in the grid environment to match the step length settings of the Bi-RRT*-Smart algorithm used in the non-grid environment. According to this method, from the results and data in Table 2 and Table 3, it can be observed that the computation time for the three A* algorithms in the grid environment is significantly less than that of the three algorithms in the previous non-grid scenario. Additionally, the path lengths are better than those achieved by the BA and GWO algorithms. Compared to the Bi-RRT*-Smart algorithm, which had the fastest time in non-grid methods, the time of the proposed improvement A* is also shorter. Therefore, we chose the grid-based method as this study’s primary method for executing path planning.

#### 3.3.2. Environment with Waypoints

**Figure 17:**
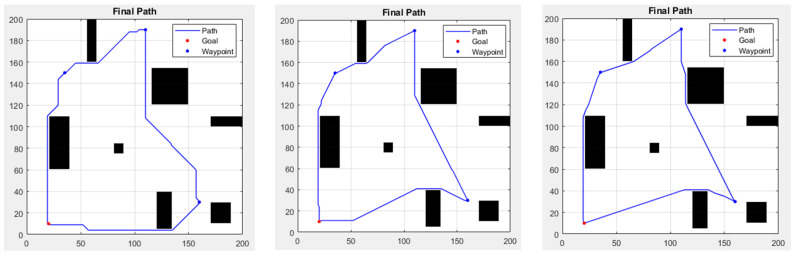
In an environment with waypoints, the results from left to right are A*-8 directions, A*-16 directions, and A*-32 directions.

To make the simulation more realistic, we have designed several waypoints to cope with mission situations that may be encountered in the real environment. In the environment set up, the red point is the starting point, and the path proceeds counterclockwise through the other blue points (the waypoints), then returning to the starting point, which is also the endpoint of the route planning. These four points are located at (20, 10), (160, 30), (110, 190), and (35, 150). Figure 17 displays the path planning results of the three methods in the waypoint environment. Table 4 shows the comparison of different algorithms in the waypoint environment.

In Figure 17, the obstacles in the first segment of the path cause three methods to choose different avoidance strategies, leading to variations in path length. Compared to the traditional A* with many turns, the improved A* has fewer noticeable turning points, resulting in a nearly direct path to the waypoints and the endpoint. In terms of results, both improved A* algorithms performed well. In some scenarios, the 16-direction version was faster; however, from a distance perspective, the 32-direction A* provided the best results among the three methods. Due to the limited direction choices, traditional A* generally lagged in speed and distance.

## 4. Obstacle Avoidance

Due to the use of hardware components like depth cameras in this study, Q-learning was selected as the basis for the obstacle avoidance system. When a drone is performing a flight mission, unforeseen situations may arise. Ignoring these potential issues and following the planned initial route can lead to dangerous situations. Therefore, local obstacle avoidance is crucial for the safety of the drone during its mission. Local obstacle avoidance involves detailed planning to navigate around both static and dynamic objects in a small area. To address this, this study has chosen a reinforcement learning algorithm that adapts well to unknown environments as the basis for obstacle avoidance. This approach will be integrated with the hardware components used in the study to achieve successful obstacle avoidance.

### 4.1. Q-Learning Algorithm

**Figure 18:**
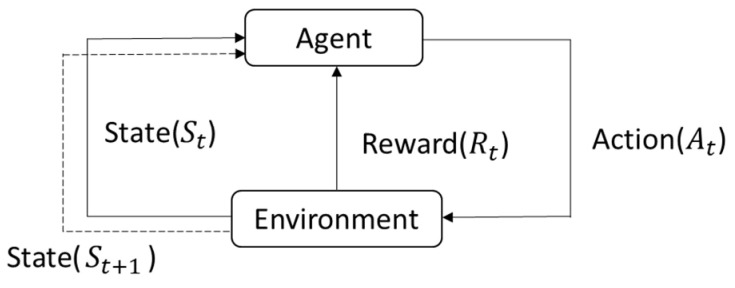
Diagram of relationship between agent and environment.

Q-learning is a type of reinforcement learning and is one of the more commonly used methods in the field [8]. It is considered foundational compared to other reinforcement learning approaches. Reinforcement learning is an algorithm that learns autonomously through interaction with its environment, without the need for explicit modeling of the environment, like in path planning algorithms. The algorithm can learn independently by setting up the appropriate functions, parameters, and specifications to find the most suitable and optimal solution. As shown in Figure 18 below, let us assume we are playing a game. The agent is the person playing the game, and the environment is the game itself. The game environment will have different states $S_{t}$ at each time t. Based on the different states $S_{t}$, the player (agent) will take different actions $A_{t}$. According to the action $A_{t}$ taken by the agent on the environment, a reward $R_{t}$ will be obtained. Then, it will proceed to the next time t+1 and get a new state, repeating the above rules until reaching the final goal or failing, at which point the operation ends. When the agent interacts with the environment, Q-learning learns from past experiences and, through different actions $A_{t}$, can maximize the accumulated reward over time. This interaction of Q-learning with the environment can be seen as a Markov Decision Process (MDP). Therefore, the algorithm aims to maximize the overall reward and learn the optimal action strategy.

**Figure 19:**
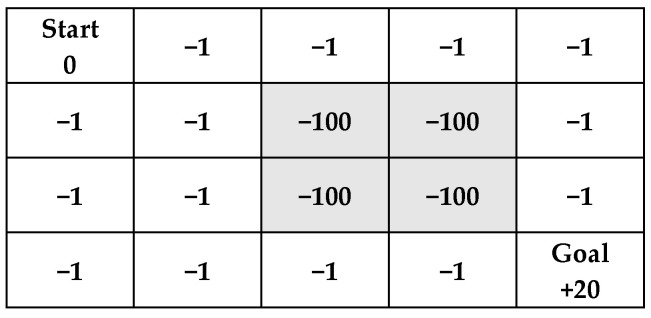
Diagram illustrating rewards in the environment, gray blocks represent obstacles.

Since we cannot wander around aimlessly while playing the game, learning from experience is very important. In Q-learning, it is necessary to set up reward rules to ensure that the results align with expectations when computing and updating Q-values. Equation (5) shows the rules established in this study. Figure 19 illustrates the reward rules in this research, where the rules can be adjusted dynamically. Rewards can be adjusted according to the problem, meaning the reward rules depend on the issue.

(5) $$Reward=\left\{\begin{array}{c} -1\\ -100\\ +20 \end{array}\right.\;\;\;\begin{array}{c} every\;step\\ enter\;the\;gray\;blocks\\ get\;the\;Goal \end{array}$$

Q-learning is a reinforcement learning method that enables an agent to learn how to make optimal decisions in various situations. It uses a “Q-table” to store the best Q-values for each state–action pair and gradually updates the Q-table based on rewards to learn the optimal strategy. A Q-table is first initialized at the initial stage of interacting with the environment. Since the environment is unfamiliar and the optimal actions are unknown, the algorithm starts by performing random actions. Exploring the unknown domain helps gather diverse information, facilitating better learning later on. Over time, decisions can be made based on the Q-table as it becomes more refined and informative through accumulated experiences. The Q-table tracks information related to the movement directions (up, down, left, right) for each state. As the agent interacts with the environment and performs actions, it receives corresponding values. These values are used in Bellman’s equation to calculate the Q-values to be stored in the Q-table. Over time, as the agent interacts more with the environment, the data in the Q-table become more refined, enabling the agent to make good decisions when encountering unknown environments. The Q-value can be derived using the following equation.

(6) $$Q^{new}\left(S_{t},A_{t}\right)=Q\left(S_{t},A_{t}\right)+\eta [r_{t}+\gamma \cdot maxQ\left(S_{t+1},A_{t+1}\right)-Q\left(S_{t},A_{t}\right)]$$

where $Q\left(S_{t},A_{t}\right)$ represents the Q-value obtained by performing action $A_{t}$ in state $S_{t}$, while $Q^{new}\left(S_{t},A_{t}\right)$ is the updated Q-value obtained using the right side of the equation. $r_{t}$ denotes the reward received after performing the action, η is the learning rate (0< η ≤ 1), and γ is the discount factor (0≤ γ ≤1). When γ is large, the agent places more emphasis on long-term rewards; conversely, when γ is small, the agent focuses more on immediate rewards. In this study, a genetic algorithm (GA) is used to select the proper γ, which can speed up the convergence process. The term $\eta [r_{t}+\gamma \cdot maxQ\left(S_{t+1},A_{t+1}\right)]$ considers both the immediate reward $r_{t}$ and the expected future reward obtained by selecting action $A_{t+1}$ in the next state $S_{t+1}$. Adding these two values yields the accumulated reward starting from the current moment. This updated strategy helps in learning the optimal policy to maximize cumulative rewards. Subtracting $Q\left(S_{t},A_{t}\right)$ represents the difference between the actual value and the predicted value, giving the error. Retaining a portion of $Q\left(S_{t},A_{t}\right)$ as 1−η ensures stable learning by not completely replacing the old estimate with the new one.

**Figure 20:**
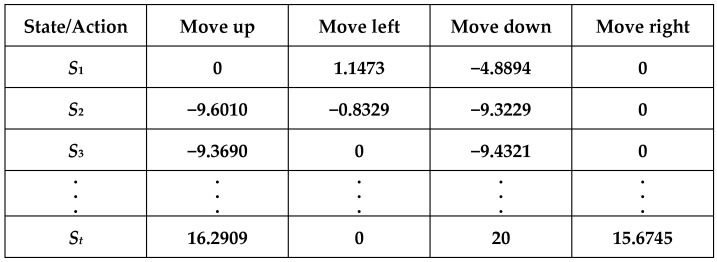
Q-table.

A Q-table is simply a lookup table that calculates the maximum reward expected after performing a specific behavior in a particular state. This table can guide us to choose the best behavior for each state. At first, we try to let the model take random actions, evaluate the reward first, and increase the Epsilon ratio to let the model do more exploratory work. Until there are enough numbers later, we can let the model take the behavior that will generate the most reward according to the records of the Q-table. This is shown in Figure 20. The data in the Q-table are obtained from the calculations based on the previous Equation (6).

### 4.2. Simulations of Obstacle Avoidance

**Figure 21:**
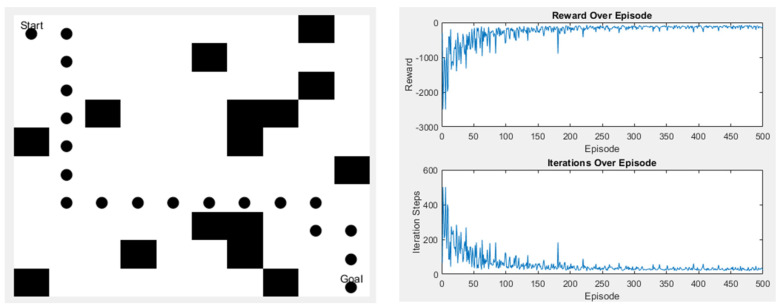
Q-learning experimental results in Scene 1.

For the algorithm to be utilized smoothly in the real environment, we set up various scenarios for the Q-learning algorithm to undergo more training and practice the effect of obstacle avoidance. First, training is conducted in a simple environment, as shown in Figure 21. In the left image, the black dots represent the path taken by Q-learning, and the black squares are obstacles. The top left corner is the starting point, and the bottom right corner is the endpoint. The right image displays the algorithm’s reward values and the number of steps taken in this scenario. The right-top graph shows the reward values, and the right-bottom graph indicates the number of steps.

**Figure 22:**
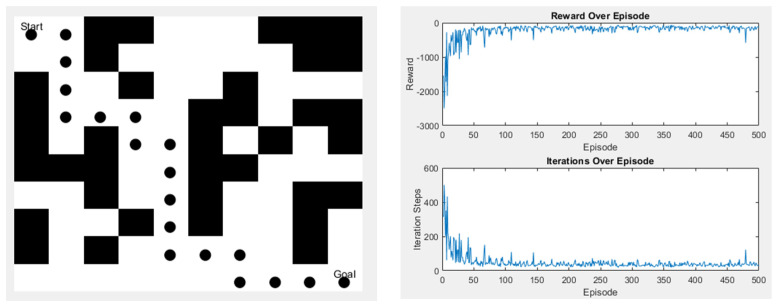
Q-learning experimental results in Scene 2.

In the second scenario, there are slightly more obstacles than in the first one, but it still has the same start and endpoints. The left image shows the path taken, while the right image displays the action values, as shown in Figure 22.

The abovementioned scenarios show that the training process initially experiences significant oscillations when there are fewer environmental obstacles. Still, the values gradually converge with more iterations. On the other hand, when obstacles occupy more of the space, the available walking area is reduced, leading to quicker convergence. However, the time spent does not differ much since all these trainings go through 500 iterations. The time spent is shown in Table 5.

**Figure 23:**
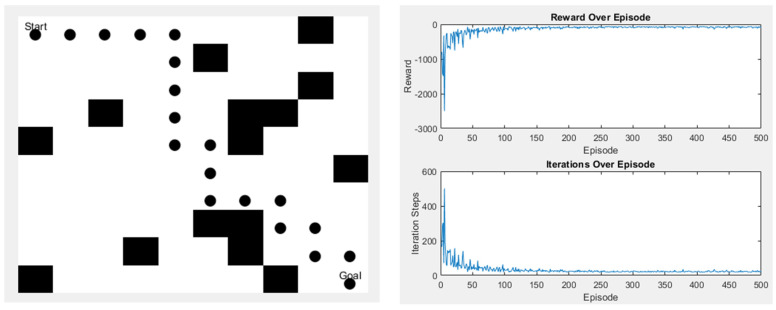
Scene 1 after parameter change.

**Figure 24:**
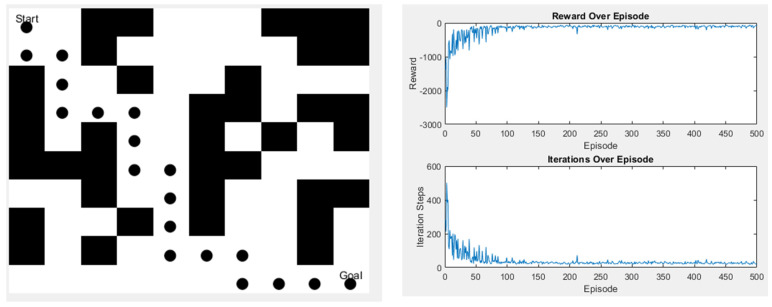
Scene 2 after parameter change.

Since the Q-learning algorithm used in this study has only four moving directions, the final path length will remain the same no matter how the paths change. However, the advantage of Q-learning is that it has many parameters, so after the initial simulation experiments, we used the genetic algorithm (GA) to find out how to make the algorithm converge faster during the iterative process in some scenarios. Although the time and the length of the algorithm remain unchanged, the increase in the convergence speed can help to minimize the waste of training time. Figure 23 and Figure 24 are schematic diagrams.

By adjusting the value of γ in Equation (6) using a GA, the adjusted reward and step count can achieve convergence earlier. The time without GA is derived from Table 4, where a common 500 iterations were used. After incorporating GA, the time reflects the results of 200 iterations. Table 6 below shows the time to convergence without GA and with GA.

## 5. Formation Flight

In recent years, the rapid development of drones has led to their use in an increasing number of fields. Drones are frequently used for large-scale tasks, and demand is gradually increasing. Thus, considering only a single drone is no longer sufficient. Multiple drones are often used in various applications such as military exercises, traditional agriculture, fisheries, and entertainment activities, making formation flying a vital topic. This is similar to the river inspection considered in this study. Since the river areas vary in size, using only one drone could waste time. Therefore, to meet the demand, multiple drones are used simultaneously. However, using multiple vehicles introduces new challenges. Due to environmental factors, situations requiring obstacle avoidance may arise, necessitating formation control to prevent the drones from falling out of formation. For example, R. Guan and G. Hu proposed a method of formation tracking using cameras [29]. In [41], a novel adaptive neural path-following control scheme in conjunction with a disturbance observer was proposed; this scheme enables an underactuated large merchant ship to perform waypoint-based path-following tasks in complex environments. In this section, we will discuss the leader–follower algorithm and its application in these scenarios.

The pilot–follower concept was first proposed by Cruz in 1978 and has been successfully applied to the formation control of mobile robots by later generations [42]. As one of the most commonly used formation control methods, its basic idea is that all the formation members are assigned to two roles: the navigator controls the movement trend of the whole formation by sailing along a predetermined or temporary path, and the follower achieves formation control by following the navigator based on the distance and orientation information relative to the navigator. The advantage of the navigator–follower method is that the formation control structure is simple and easy to implement, and only the navigator’s desired path or other behavior needs to be set in the formation. Then, the follower follows the navigator with a predetermined positional offset to realize the formation control. For this reason, the pilot–follower method is widely used in practical engineering. The disadvantage of this method is that the formation system is too dependent on the navigator, which may cause the whole group of UAVs to pay a price if there is an unexpected situation.

### 5.1. Graph Theory

Formation flying primarily consists of a leading drone and follower drones. The leading drone’s main task is to follow the path, while the follower drone’s main task is to follow the leader. An organization with many drones may have more than one leading drone. However, three drones are used in this river patrol, so only one leader exists. The primary method used to calculate the positions of the drones in this formation flight is the Laplacian matrix and system equations [43].

In graph theory, we can differentiate between the degree and adjacency matrices. The degree matrix is a diagonal matrix representing the number of edges connected to each node, given by the following formula.

(7) $$D_{i,j}=\left\{\;\begin{array}{c} dev\left(v_{i}\right),\;if\;i=j\;\;\;\;\;\\ 0,\;otherwise\;\; \end{array}\right.$$

The adjacency matrix is a symmetric matrix with a diagonal of 0. It represents the data transfer between each point and is given by the following equation.

(8) $$A_{i,j}=\left\{\;\begin{array}{c} 1,\;if\;j\;transmit\;to\;i\;\;\;\;\\ 0,\;otherwise\;\;\;\;\;\;\;\;\;\;\;\;\;\;\;\;\; \end{array}\right.$$

The formula for the Laplacian matrix is as follows.

(9) $$L=D-A=\left\{\begin{array}{c} deg\left(v_{i}\right),\;if\;i=j\\ -1,\;otherwise \end{array}\right.$$

**Figure 25:**
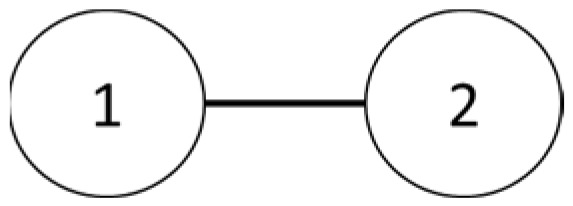
Two-drone structure.

**Figure 26:**
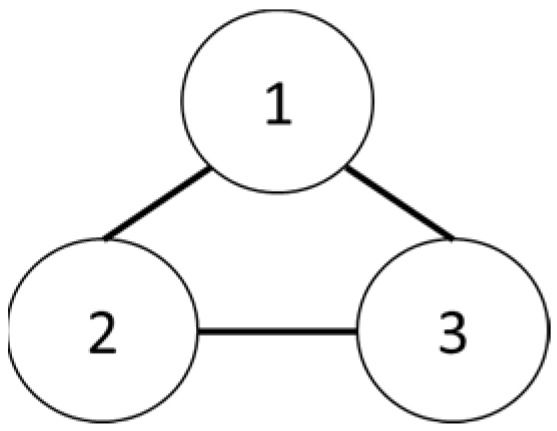
Three-drone structure.

The team is organized as seen in Figure 25 and Figure 26.

### 5.2. Formation Control

Referring to the information provided in [43], it is assumed that the drones all have the same properties.

(10) $${\dot{x}}_{i}=Ax_{i}+Bu_{i}\;\;,\;i=1,\ldots ,N$$

where $x_{i}$ represents the overall shape of the drone, which contains the position and speed, $u_{i}$ represents the input control commands, which are used to maintain the formation shape, and A and B are the set matrices, as shown in the following equation. A represents the speed and position of the drone, B controls the formation position of the follower, and n is the number of drones.

(11) $$\left\{\begin{array}{c} \;\;A=\left[\begin{array}{cc} 0 & 1\\ 0 & 0 \end{array}\right]\;\\ \;\;B=\left[\begin{array}{cc} 0 & 0\\ 1 & 1 \end{array}\right]\; \end{array}\right.$$

Kronecker tensor processes A and B are used to control velocity and acceleration separately.

(12) $$\left\{\;\;\;\begin{array}{c} A_{d}=I_{{\left(2N\right)}^{2}}+0.1*I_{N^{2}}⊗A\\ B_{d}=0.1*L⊗B\;\;\;\;\;\;\;\;\;\;\;\;\;\;\;\;\;\; \end{array}\right.$$

where I is the unit matrix, N is the number of drones, $A_{d}$ and $B_{d}$ are the system equations, ⊗ is the Kronecker tensor product, and L is the Laplace matrix.

Enter the position, speed, and formation to be displayed in the formula shown below for three drones as an example.

(13) $$P(t)=\left[\begin{array}{ccc} \begin{array}{cc} \begin{array}{c} P_{x_{1}}\\ P_{y_{1}} \end{array} & \begin{array}{c} V_{x_{1}}\\ V_{y_{1}} \end{array} \end{array} & \begin{array}{ccc} \begin{array}{c} P_{x_{2}}\\ P_{y_{2}} \end{array} & \begin{array}{c} V_{x_{2}}\\ V_{y_{2}} \end{array} & \begin{array}{c} P_{x_{3}}\\ P_{y_{3}} \end{array} \end{array} & \begin{array}{c} V_{x_{3}}\\ V_{y_{3}} \end{array} \end{array}\right]F=\left[\begin{array}{ccc} \begin{array}{cc} \begin{array}{c} F_{x_{1}}\\ F_{y_{1}} \end{array} & \begin{array}{c} 0\\ 0 \end{array} \end{array} & \begin{array}{ccc} \begin{array}{c} F_{x_{2}}\\ F_{y_{2}} \end{array} & \begin{array}{c} 0\\ 0 \end{array} & \begin{array}{c} F_{x_{3}}\\ F_{y_{3}} \end{array} \end{array} & \begin{array}{c} 0\\ 0 \end{array} \end{array}\right]$$

where P(t) is the position and velocity information of each drone, and F represents the formation of the drone. ${P_{x}}_{i}$ and ${P_{y}}_{i}$ are the position of the $i^{th}$ drone; the speed of the $i^{th}$ drone can be expressed as $V_{x_{i}}$ and $V_{y_{i}}$, which is usually a fixed value. ${F_{x}}_{i}$ and ${F_{y}}_{i}$ represent the position of $i^{th}$ drone in the formation.

Bringing the position and formation into the formula calculates the speed and path of the leader drone, which in turn can calculate the speed and next-moment position of the other follower drones. The formula is as follows.

(14) $$P\left(t+1\right)=A_{d}\times P\left(t\right)+B_{d}\times (P(t)-F)$$

By using these formulas, we are able to operate the drone smoothly, making it possible to keep the formation so that the follower can follow the leader drone well enough to conduct experiments.

### 5.3. Simulation Results

**Figure 27:**
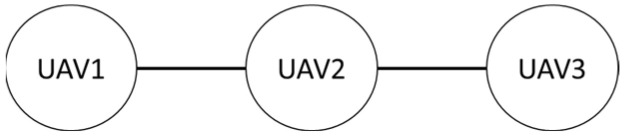
Row queue.

**Figure 28:**
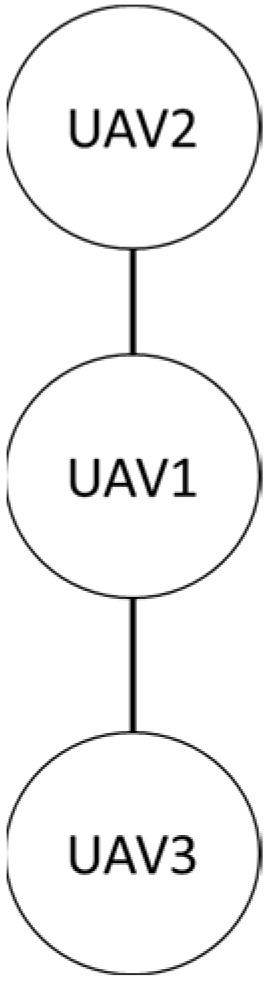
Column queue.

Firstly, in the absence of obstacles, the formation shape used by the three drones in this study is shown. The first is the row formation, and the second is the column formation, schematically shown in Figure 27 and Figure 28. UAV2 is the leader in the system, and UAV1 and UAV3 are the followers.

**Figure 29:**
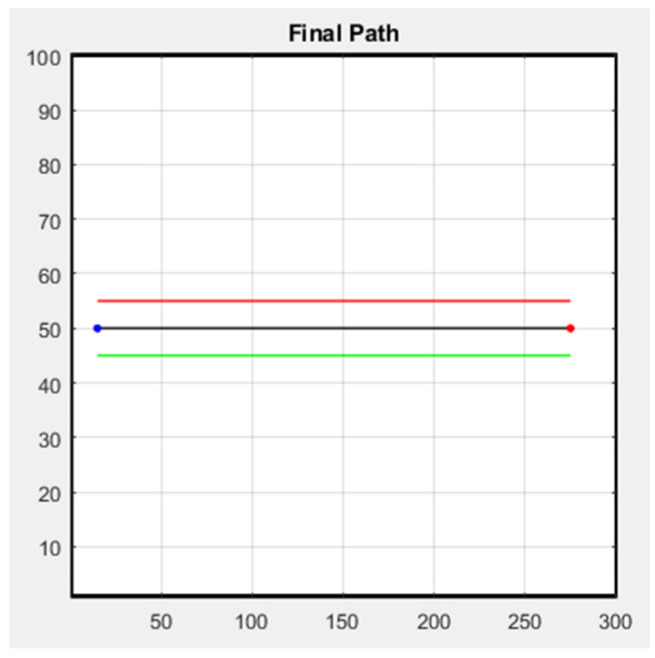
Row simulation.

**Figure 30:**
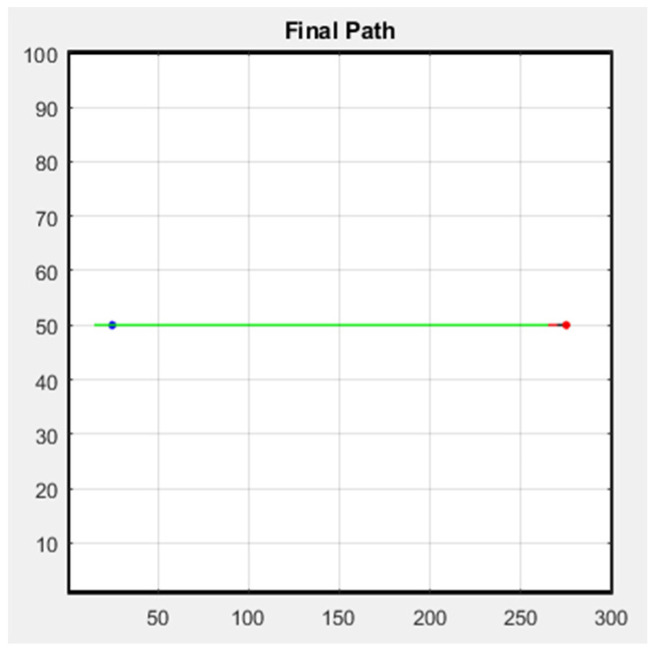
Column simulation.

The following figure shows the simulation result of the formation; Figure 29 is the row formation, and Figure 30 is the column formation. The leader’s starting point is at (15, 50) and the ending point is at (275, 50) in Figure 29 and Figure 30. For the other two followers, we keep them at a distance of 5 m from the leader to stay safe. The blue line is the leader’s trajectory, and the red and green lines are the followers’ trajectories.

**Figure 31:**
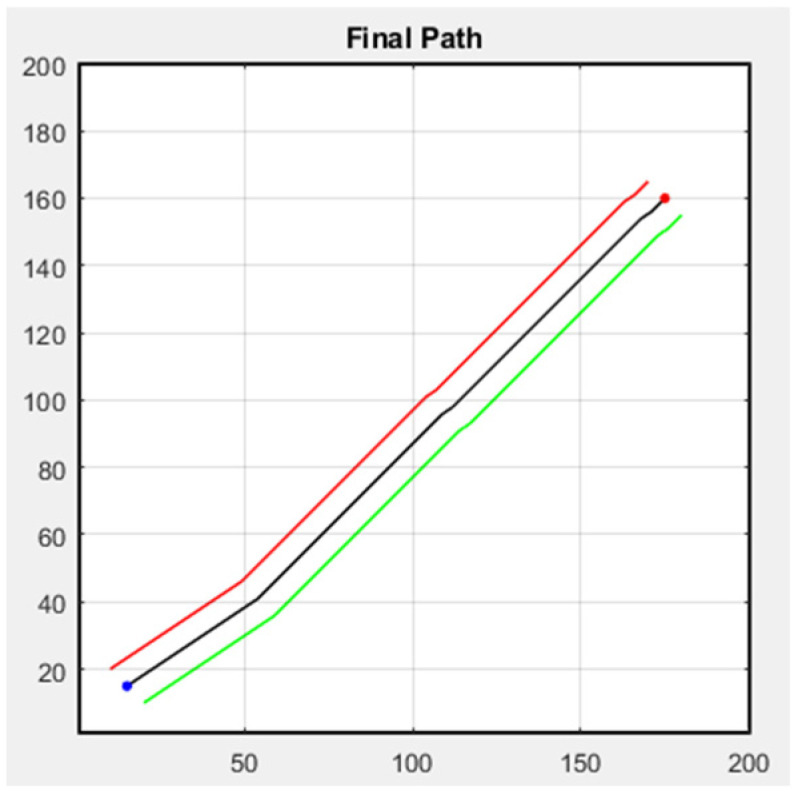
Formation flying without obstacles.

**Figure 32:**
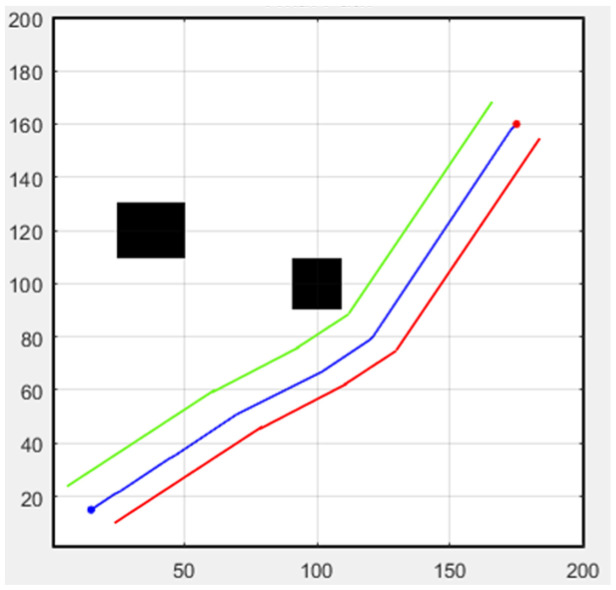
Formation flying with static obstacles (black boxes).

Next, we discuss formation flight combined with path planning. Here, we use a 32-direction A* algorithm, which has been improved upon in this study. First, formation flying is performed in an environment without obstacles, with the simulation results shown in Figure 31. The second scenario is when obstacles appear in the environment. Besides following the original path, the additional drone on the left must successfully avoid obstacles, as shown in Figure 32. In both scenarios, the starting point is (15, 15), and the endpoint is (175, 160).

**Figure 33:**
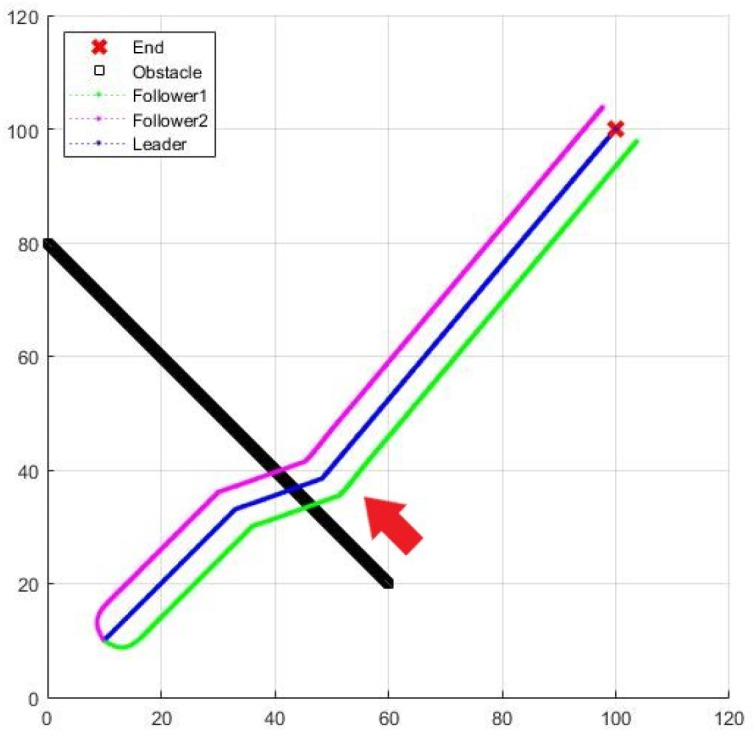
Formation flying with a dynamic obstacle.

**Figure 34:**
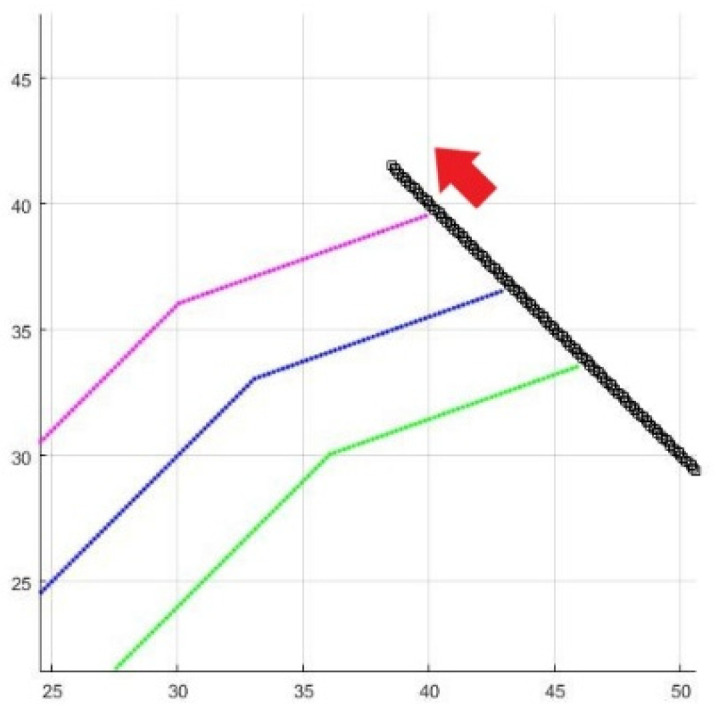
Enlarged formation of Figure 33.

A dynamic obstacle is added to the three-UAV formation flight. Figure 33 and Figure 34 are the result and process of avoiding the dynamic obstacle, where the black line is the trajectory of the dynamic obstacle, and the red arrow is its moving direction.

## 6. Field Tests

**Figure 35:**
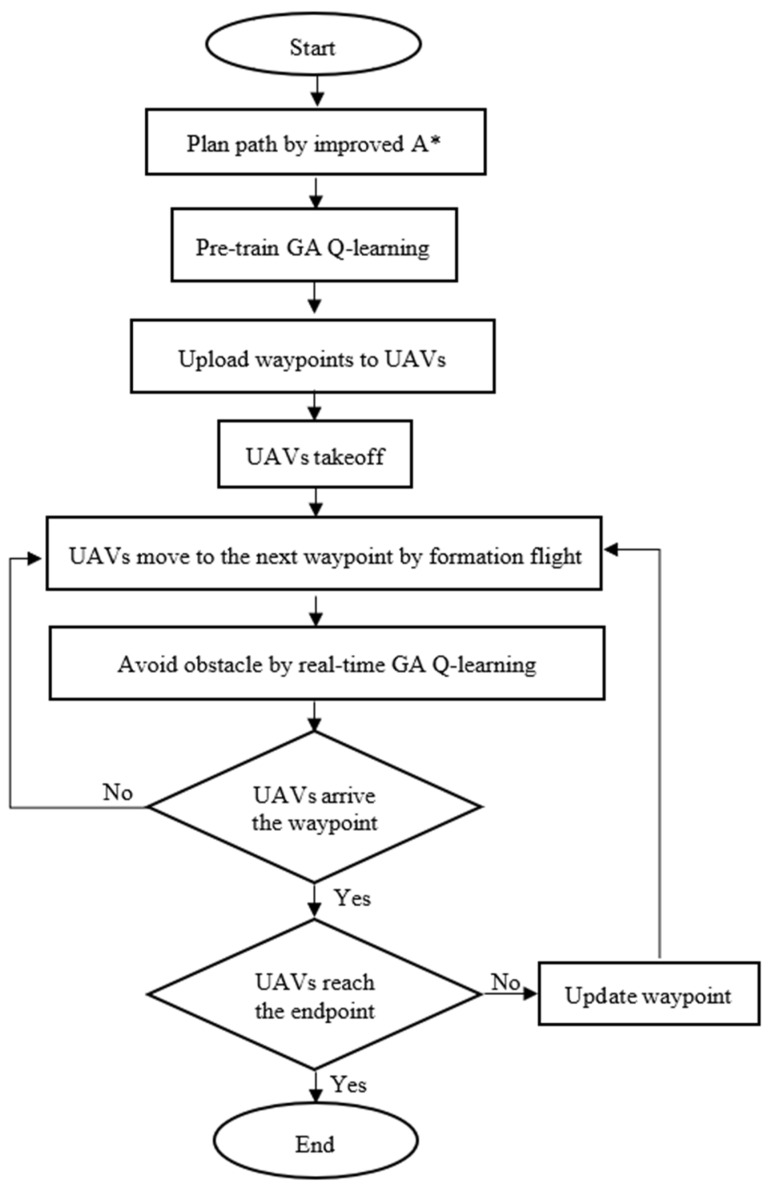
Flowchart of the sequence of the proposed method.

In this section, we apply the simulations from previous sections to real drones. We installed the onboard computer Jetson AGX Xavier on the drones and used it to compute mission tasks. The Jetson AGX Xavier was integrated with the flight control board, Intel RealSense D415 depth camera, and GPS receiver to conduct drone path planning, obstacle avoidance, and formation flying experiments. Due to the extensive equipment required for three drones, it was inconvenient to transport them outdoors, so the experiments were conducted within the school premises. A flowchart of the sequence of steps of the proposed method is shown in Figure 35.

### 6.1. Assumptions

The problem setup was based on the following assumptions and physical constraints. The speed and heading angle limitations of the UAV are 4 m/s and 65 degrees, respectively. The flight time of the UAV is limited to 20 min. Wind speed is less than 5 m/s. The number of degrees of freedom applied to the UAV is 4. The workstation for this study is the Jetson AGX Xavier. The GPU is NVIDIA Volta with 512 NVIDIA CUDA cores and 64 Tensor cores. The CPU is an 8-core NVIDIA Carmel Armv8.2 64-bit (NVIDIA, Santa Clara, CA, USA).

### 6.2. Obstacle Avoidance and Formation Flight

**Figure 36:**
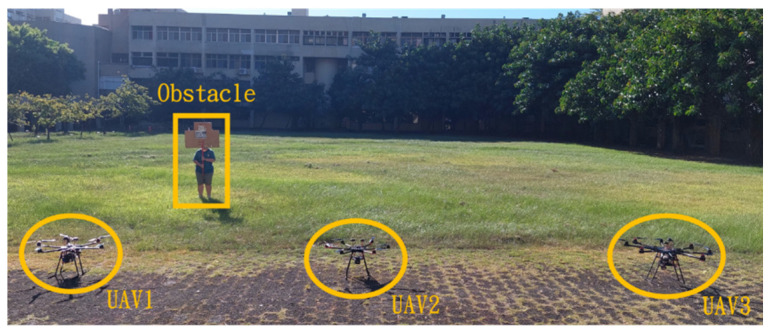
Obstacle condition-first scenario.

**Figure 37:**
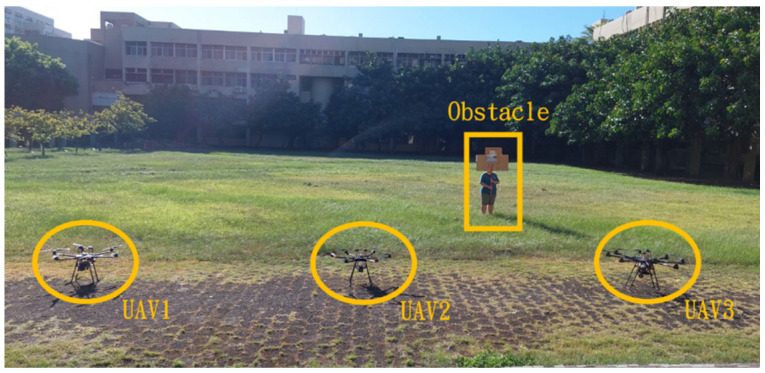
Obstacle condition-second scenario.

This section mainly demonstrates drone path planning for obstacle avoidance and formation flying when the number of drones exceeds two. The scenario involves using three drones for path planning and formation control. In formation flight, the distance of each UAV from its neighbor UAV is about 2 m. The first situation is when the obstacle appears directly in front of the left-side drone; the second is when the obstacle is in front of the right-side drone. Figure 36 and Figure 37 illustrate these two types of obstacle conditions.

**Figure 38:**
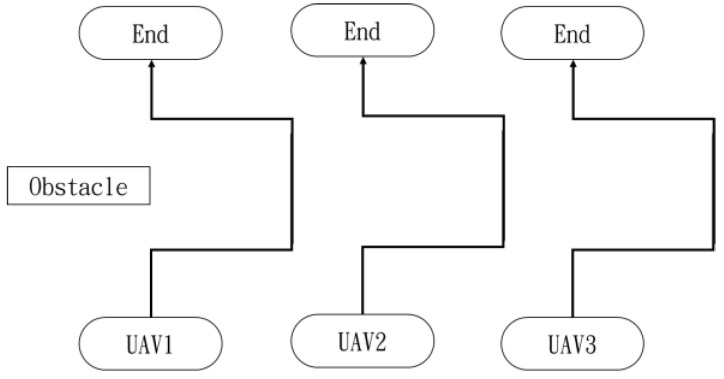
Drone path schematic when the obstacle appears on the left side.

**Figure 39:**
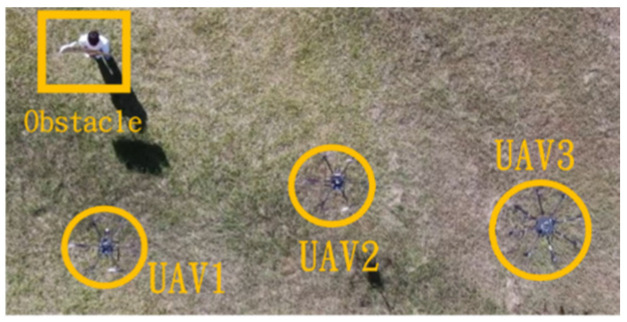
Avoidance process one when the obstacle appears on the left side.

**Figure 40:**
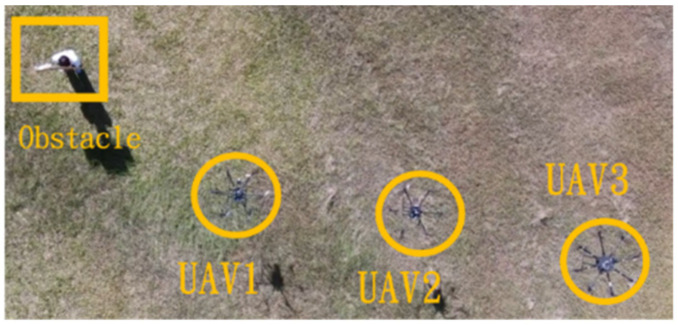
Avoidance process two when the obstacle appears on the left side.

**Figure 41:**
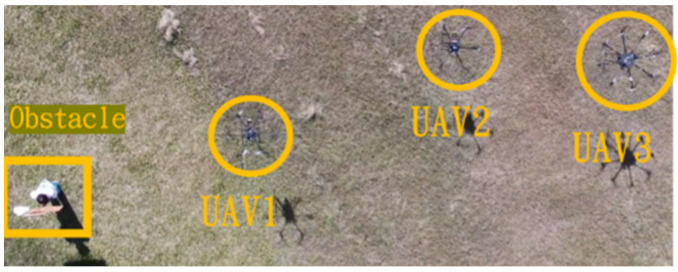
Avoidance process three when the obstacle appears on the left side.

**Figure 42:**
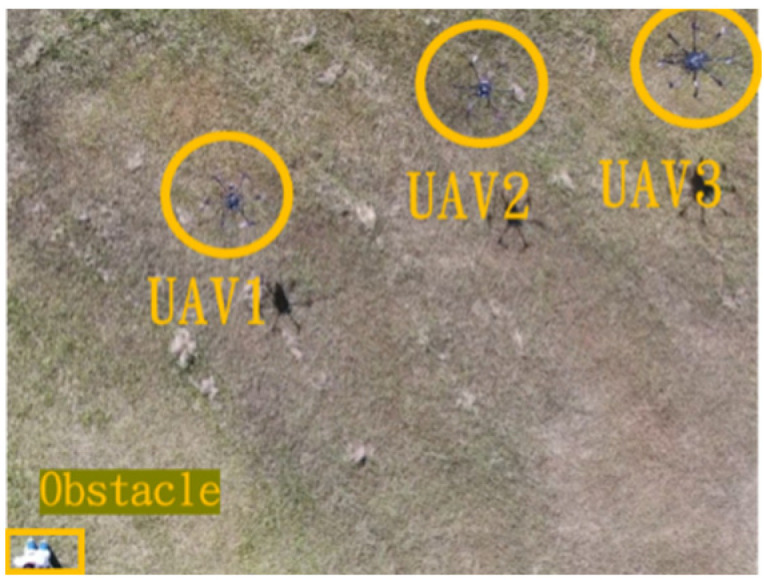
Avoidance process four when the obstacle appears on the left side.

In the first situation, the path planning schematic is shown in Figure 38. When the obstacle appears on the left side, the drones will collectively maneuver to the right to avoid it. Figure 38, Figure 39, Figure 40, Figure 41 and Figure 42 depict the flight results.

**Figure 43:**
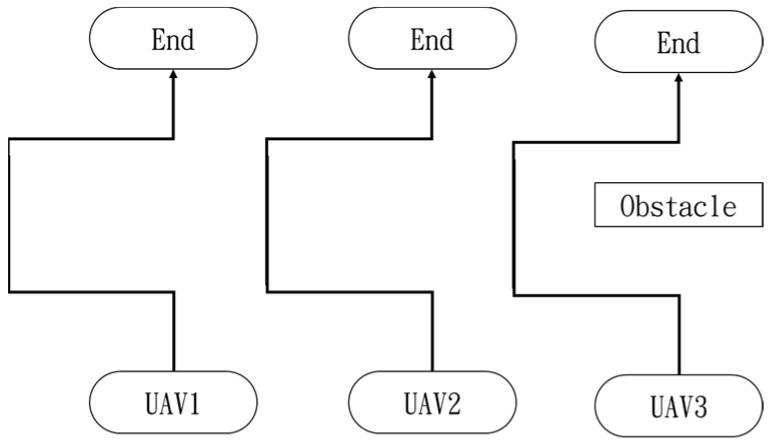
Drone path schematic when the obstacle appears on the right side.

**Figure 44:**
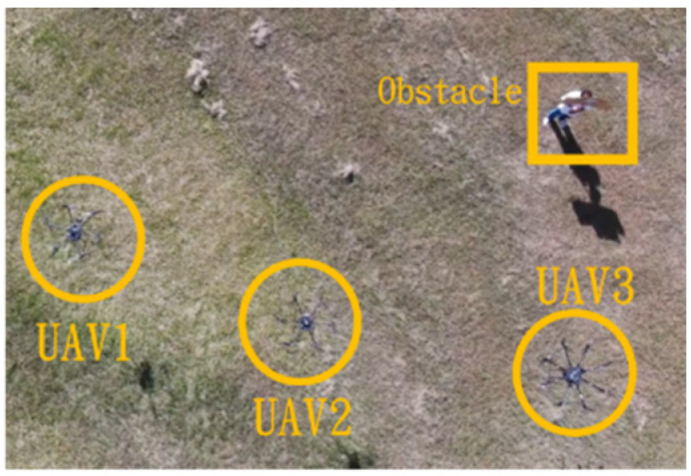
Avoidance process one when the obstacle appears on the right side.

**Figure 45:**
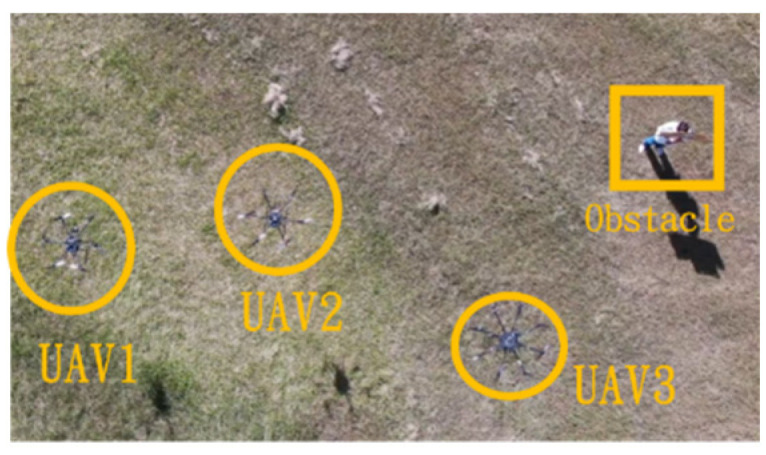
Avoidance process two when the obstacle appears on the right side.

**Figure 46:**
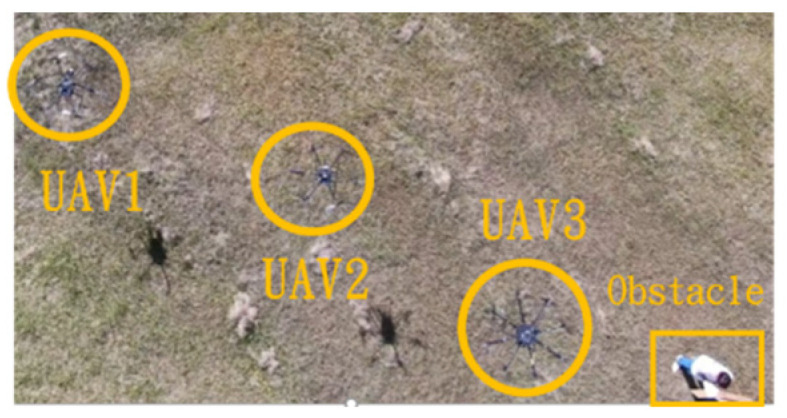
Avoidance process three when the obstacle appears on the right side.

**Figure 47:**
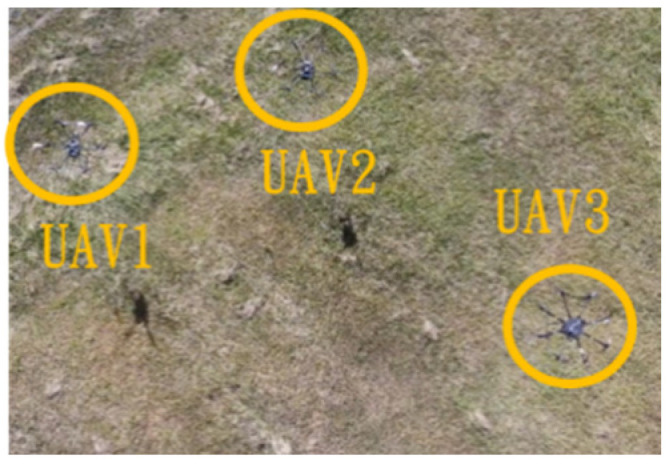
Avoidance process four when the obstacle appears on the right side.

In the second situation, when the obstacle appears on the right side, the drones will collectively maneuver to the left to avoid it. The flight process is illustrated in Figure 43, Figure 44, Figure 45, Figure 46 and Figure 47.

### 6.3. Formation Flight in Waypoint Environment

**Figure 48:**
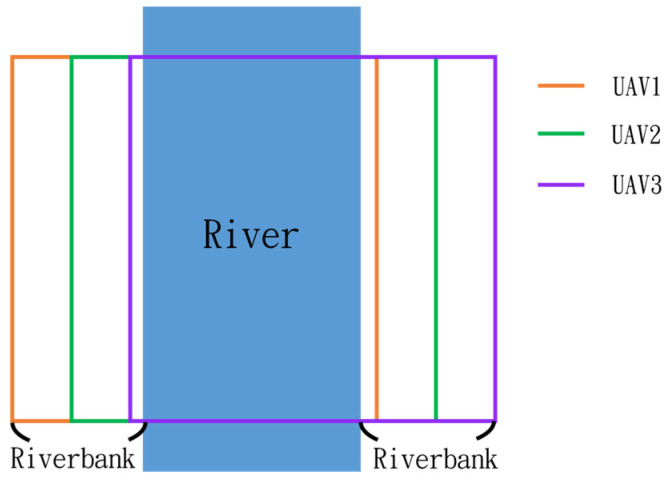
Riverbank inspection routes of three UAVs.

In this simulation, we model the scenario of flying over an imaginary river (Figure 48). Four waypoints are placed at the four corners of a rectangular form to allow the drones to plan their route.

**Figure 49:**
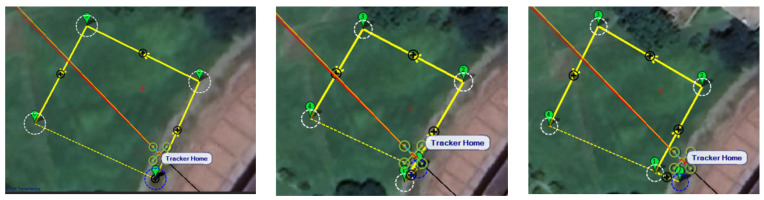
From left to right: planned routes of UAV-1, UAV-2, and UAV-3.

**Figure 50:**
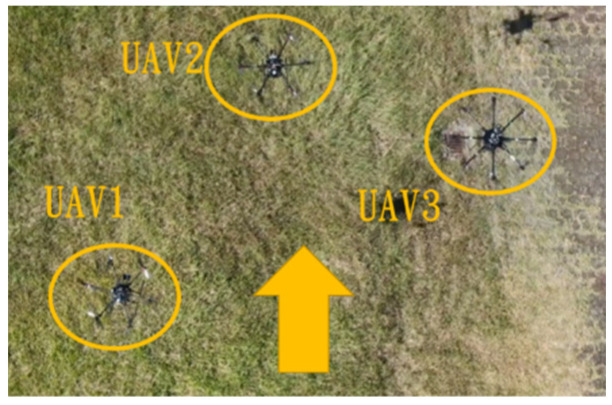
Fly to waypoint one.

**Figure 51:**
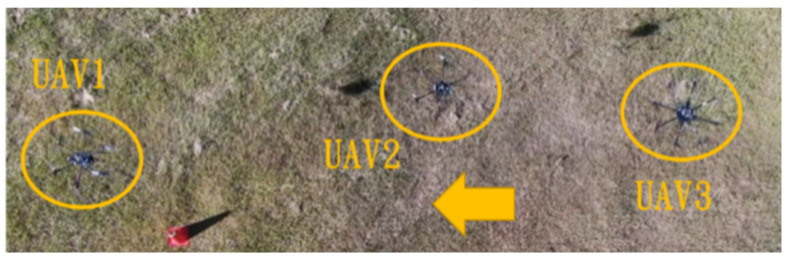
Fly to waypoint two.

**Figure 52:**
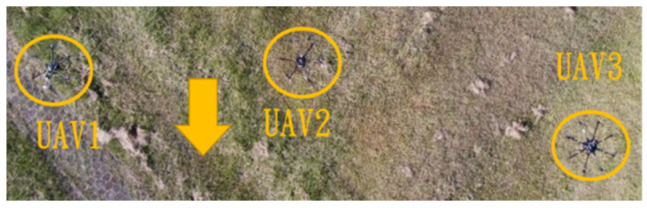
Fly to waypoint three.

**Figure 53:**
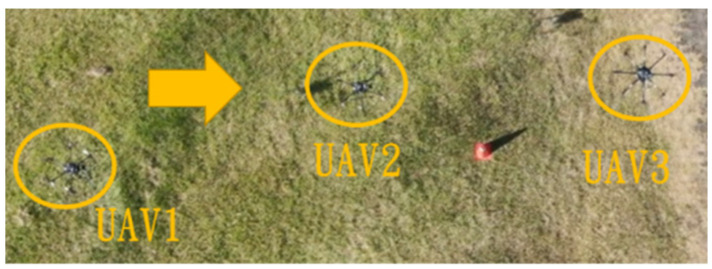
Fly to waypoint four.

Since inspection is needed along the river, initially, three UAVs fly horizontally to waypoints one and three. When crossing the river, three UAVs fly in a column formation to waypoints two and four. Figure 49 shows the predefined route, while Figure 50, Figure 51, Figure 52 and Figure 53 depict the actual flight process. In Figure 49, the yellow line is the planned path, the red line is the heading direction of each UAV at the starting point, and the green marks are the waypoints.

**Figure 54:**
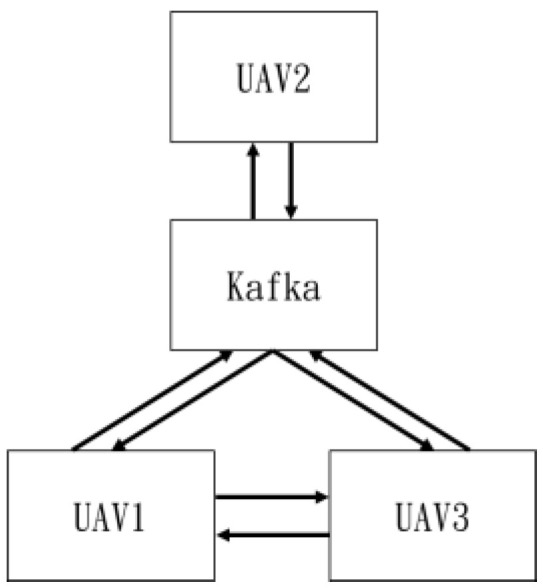
Data transmission.

In the flight control of multiple drones mentioned above, besides formation flying, Kafka was used for information transmission [44]. Figure 54 illustrates the transmission diagram.

## 7. Conclusions

This study primarily focuses on path planning, obstacle avoidance, and formation flight for riverbank inspection missions. By combining the improved A*, Laplacian matrix, and Q-learning methods, multiple UAVs can effectively perform inspection tasks. Initially, this study uses the improved A* algorithm for waypoint planning across the entire environment. Regarding path planning, the A* algorithm is improved using a grid-based approach and has thirty-two moving directions, whereas the conventional A* has only four directions. This improvement enables the proposed A* method can search more directions in path planning and obtain a more optimal path than the conventional A* method. For obstacle avoidance, depth cameras are installed on the drones, and the obtained images are processed by reinforcement Q-learning with a genetic algorithm to navigate around obstacles. In Q-learning algorithm, there is a key factor that needs to be selected. Conventionally, this factor is selected by trial and error. In this study, we applied GA to reinforcement learning to select the optimal value for the method and shorten the learning process. Compared to the traditional A*, the improved A* proposed in this study shortens both the planning path and time, enhancing overall efficiency. When encountering obstacles in the environment, the GA Q-learning method, known for its adaptability, successfully avoids obstacles. For controlling multiple UAVs, the Laplacian matrix ensures that the UAVs maintain formation flight until they reach the destination. In real-world experiments, Kafka’s data transmission synchronizes the UAVs, allowing them to perform flight tasks more coherently. During this study, our self-assembled drones frequently encountered interference from wind and magnetic fields due to environmental factors. In the experiments, the drones were often battered by strong winds, causing them to wobble back and forth and sway side to side, leading to instability. Wind interference would cause the drones to deviate from their original paths, resulting in significant discrepancies between the actual outcomes and the simulation results. Combining the advantages of the flight control board and the GPS receiver to address wind resistance could provide drones with a robust positioning system and enhanced wind resistance capabilities. During fixed periods of drone operation on campus, we frequently experienced connectivity issues between the computer and the drones. For magnetic interference, we currently use a three-axis geomagnetic sensor to monitor the surrounding magnetic field. However, this only provides a short-term solution, not a long-term fix. During the drone formation flights, the initial intent was to enhance synchronization and command transmission between the drones. However, due to Kafka’s reliance on network transmission, the time delay in data transmission caused discrepancies in information between the drones, leading to disarray in their formation. Utilizing better communication equipment could potentially speed up data transmission. Alternatively, adding extra equipment to enable direct communication between the drones might enhance the effectiveness of their flying formation. In future works, we will add LiDAR or sonar sensors to enhance the ability for collision avoidance. To overcome higher wind speed disturbance, the easiest way is to set up a large-scale UAV that can withstand wind speeds up to level 5. A wind field simulation model is also good for UAV operation, which can provide approximate wind field environments before the UAV performs its missions.

## Figures and Tables

**Table 1:** Comparison of different methods.

Method	Optimal Path Length	Computing Time
Bi-RRT*-Smart	short	moderate
BA	long	long
GWO	long	moderate
PSO	short	moderate
D*	moderate	short
A*	moderate	short
Improved A*	short	short

**Table 2:** Comparison of different algorithms in a non-grid environment.

Method	Length (m)	Time (s)
Bi-RRT*-Smart	556.31	1.6341
BA	668.77	4.7884
GWO	644.43	2.7697

**Table 3:** Comparison of different algorithms.

Method	Length(m)	Time(s)
A*-8 directions	578.555	0.355
A*-16 directions	567.865	0.485
A*-32 directions	563.583	0.657

**Table 4:** Comparison of different algorithms in waypoint environment.

Method	Length (m)	Time (s)
A*- 8 directions	573.161	0.4469
A*- 16 directions	553.203	0.4035
A*- 32 directions	548.104	0.4220

**Table 5:** Q-learning time spent training in the environment.

Environment	Time (s)
Scene 1	0.5321
Scene 2	0.4605

**Table 6:** Comparison of convergence speed.

Environment	Without GA	With GA
Scene-1	0.5321	0.3864
Scene-2	0.4605	0.3711

## Data Availability

Data are contained within the article.
